# K-Means Based Bee Colony Optimization for Clustering in Heterogeneous Sensor Network

**DOI:** 10.3390/s24237603

**Published:** 2024-11-28

**Authors:** Prince Modey, Gaddafi Abdul-Salaam, Emmanuel Freeman, Patrick Acheampong, William Leslie Brown-Acquaye, Israel Edem Agbehadji, Richard C. Millham

**Affiliations:** 1 Department of Computer Science, Ho Technical University, Ho VH-0044, Ghana; 2 Department of Computer Science, Ghana Communication Technology University, Accra PMB 100, Ghana; pacheampong@gctu.edu.gh (P.A.); wbrown@gctu.edu.gh (W.L.B.-A.); 3 Department of Computer Science, Kwame Nkrumah University of Science and Technology, Kumasi K384, Ghana; gaddafi.ict@knust.edu.gh; 4 Faculty of Accounting Durban, University of Technology, P.O. Box 1334, Durban 4000, South Africa; israeldel2006@gmail.com; 5 ICT and Society Research Group, Department of Information Technology, Durban University of Technology, P.O. Box 1334, Durban 4000, South Africa; richardm1@dut.ac.za

**Keywords:** wireless sensor networks (WSNs), bee colony optimization (BCO), nature-inspired, optimization, clustering

## Abstract

In Wireless Sensor Networks (WSNs), an efficient clustering technique is critical in optimizing the energy level of networked sensors and prolonging the network lifetime. While the traditional bee colony optimization technique has been widely used as a clustering technique in WSN, it mostly suffers from energy efficiency and network performance. This study proposes a Bee Colony Optimization that synergistically combines K-mean algorithms (referred to as K-BCO) for efficient clustering in heterogeneous sensor networks. This is to develop a robust and efficient clustering algorithm that addresses the challenges of energy consumption and network performance in WSNs. The K-BCO algorithm outperformed comparative clustering algorithms such as H-LEACH, DBCP, and ABC-ACO in average error rate (AER), average data delivery rate (ADDR), and average energy consumption (AEC) for transmitting data packets from sensors to cluster heads. The K-BCO outperformed other algorithms in terms of ADDR at 95.00% against H-LEACH (75.86%), DBCP (72.07%) and ABC-ACO (90.08%). The findings indicate that the K-BCO not only optimizes energy consumption but also guarantees more stable and robust solutions, thereby extending the network lifetime of WSNs. Thus, K-BCO is recommended to practitioners in wireless sensor networks as it paves the way for more efficient and sustainable wireless communication.

## 1. Introduction

Wireless Sensor Network (WSN) comprises independent sensors deployed across a geographical area to collect and share data or information through wireless communication channels [1]. These sensors are deployed to observe physical or environmental properties like vibration, pressure, temperature, sound, motion, or pollutants. The sensors can collaborate to transmit data to a central point within the network known as the sink node (SN) or base station (BS) [2]. Wireless Sensor Networks (WSNs) provide many benefits in various applications, such as environmental monitoring, disaster management, healthcare, agriculture, and smart city infrastructure. This makes them valuable technology in different fields, including real-time data collection, enhanced monitoring and control, increased safety and security, and innovative applications [1,3]. Although WSNs are valuable in these areas, their successful development and deployment are faced with many challenges, including power consumption and packet loss. Sensors are typically battery-powered, and energy is a critical resource. Once a sensor node’s battery is depleted, it can no longer function, leading to gaps in data collection and potentially compromising the entire network. In WSN, sensors may have different energy requirements. As the number of nodes increases, the volume of data transmitted can lead to network congestion and increased energy consumption. Efficient data routing and transmission strategies are essential to mitigate these issues. The lifetime of a WSN is often limited by the energy of its nodes. Therefore, strategies that prolong the operational time of the network are crucial for maintaining continuous monitoring and data collection. However, the challenge is selecting a sensor that has enough energy to be the Cluster Head (CH).

Clustering in WSNs provides a structured technique for WSN organization, resource management, and communication optimization, leading to improved scalability, efficiency, reliability, and performance of wireless sensors [2,4]. Clustering is a method used to group sensor nodes into clusters, where each cluster has a designated leader known as the cluster head (CH). This approach reduces the amount of data that needs to be transmitted to the sink node, as data from individual nodes can be aggregated at the cluster head before being sent. While clustering can enhance energy efficiency and data management, traditional methods may not effectively adapt to the dynamic nature of WSNs, especially when considering the varying energy levels and capabilities of different sensor nodes. Again, it helps WSNs to optimize energy consumption through the Cluster Head, Duty Rotation, Data Aggregation, Efficient Routing, Sleep Scheduling, and Localized Processing [2].

Nature-inspired optimization methods are one of the approaches to solving wireless sensor network problems in terms of finding the optimal approach to cluster formation. Nature-inspired approaches help avoid unpromising search paths in any optimization problem, thus helping reduce the time spent searching for the best or near-optimal solutions. Examples of such methods include Bee Colony Optimization (BCO), which is a nature-inspired optimization algorithm based on honeybees’ foraging behavior [5,6]. BCO is a metaheuristic optimization algorithm inspired by the foraging behavior of honeybees. It mimics how bees search for food and communicate the location of food sources to one another. BCO can be used to optimize routing and clustering by efficiently selecting cluster heads based on factors like energy levels and node proximity. The foraging behavior of honeybees could be applied to find the most efficient routes for data transmission in a WSN, thereby reducing energy consumption, improving network lifetime, and enhancing the overall network performance [3].

Unfortunately, the traditional Bee Colony Optimization (BCO) faces challenges such as premature convergence, where the algorithm settles on suboptimal solutions too quickly, limiting its effectiveness in complex scenarios [5]. This also limits its performance in finding the optimal routing paths in heterogeneous sensor networks. This is because there is diversity and variability among the sensor nodes within the network. This study focuses on developing an efficient clustering algorithm by combining the k-mean clustering technique with the Bee Colony Optimization algorithm (K-BCO) to address the challenge associated with the traditional BCO technique. K-means is a popular clustering algorithm that partitions data into K clusters based on similarity [7]. It is straightforward and efficient but can struggle with dynamic environments and may not account for the varying energy levels of sensor nodes in WSNs.

In a heterogeneous WSN, sensors may have varying hardware specifications, memory capacity, processing power, and energy resources [8]. For instance, some sensor nodes might have strong batteries or an efficient sensing capability, while others may be more limited. This variation affects how sensor nodes participate in data collection, processing, and communication. Different sensor nodes may generate data at varying rates depending on their sensing capabilities and the environmental conditions they monitor. This variability can lead to uneven data traffic within the network, complicating the clustering and data aggregation processes. The proposed K-BCO algorithm aims to account for these differences to enhance data collection efficiency. The study also considers the importance of energy efficiency in WSNs. Heterogeneity in energy levels among sensor nodes means that some nodes may have more energy available for data transmission and processing than others. The K-BCO algorithm is designed to select cluster heads based on their energy status, ensuring that nodes with sufficient energy are chosen to lead data transmission. This approach helps to balance energy consumption across the network, prolonging the overall network lifetime. The physical arrangement of sensors can change over time, especially in situations where mobile sensors are involved. The K-BCO algorithm’s ability to dynamically adjust cluster formations helps maintain connectivity and communication efficiency in such environments. WSNs have unique requirements regarding data accuracy, latency, and reliability. The heterogeneity of application needs means that a one-size-fits-all clustering approach may be ineffective. The K-BCO algorithm is designed to be adaptable, allowing it to cater to the specific demands of various applications while optimizing energy consumption and network performance.

The sections of this paper are organized as follows: Section 2 (related works), Section 3 (theoretical framework), Section 4 (proposed clustering technique), Section 5 (experimental settings of WSN), Section 6 (simulation results), Section 7 (discussion of results), Section 8 (conclusions).

## 2. Related Works

Jacob I. and Darney P. (2021) [9] developed the Artificial Bee Colony (ABC) Optimization Algorithm for enhancing routing in wireless networks. The Artificial Bee Colony (ABC) Optimization aimed at improving the performance and efficiency of wireless communication systems by applying nature-inspired optimization techniques. Comparative analysis of network performance was performed using performance metrics such as packet delivery ratio, latency, and energy efficiency between the ABC algorithm and existing models. The study underscores the significance of nature-inspired optimization algorithms, particularly the ABC algorithm, in improving routing efficiency, reducing interference, and enhancing overall network performance in wireless communication systems. It stated potential applications of the developed algorithm in addressing complex challenges and optimizing wireless networks for various technological advancements. Although the study enhanced the performance of the entire network, the proposed scheme was unable to select optimal paths based on dynamic network conditions and traffic patterns.

Dawood et al. (2021) [10] also proposed an algorithm for energy-efficient clustering in heterogeneous wireless sensor networks. The algorithm was developed to improve energy efficiency in terms of intra-cluster communication by considering distance and energy factors. Their study aimed at reducing energy consumption within clusters by minimizing communication distances between nodes and cluster heads. The key components of the proposed algorithm include cluster formation, cluster head selection based on proximity and energy levels, and optimizing intra-cluster communication to reduce energy consumption. They presented simulation results, comparing the performance of the proposed protocol with the PEGASIS protocol in terms of PDR, average end-to-end delay, and energy consumption. The proposed algorithm outperformed the PEGASIS protocol in terms of lifetime, energy efficiency, and average end-to-end delay, thereby improving the lifetime of the wireless sensor network. Also, using proximity to the center of the zone and maximum residual energy as criteria for CH selection may affect the overall performance of the network since a faulty sensor may have more energy. This may disrupt effective data collection from some segments of the field. The proposed algorithm did not consider mobile sensors within the network, hence raising the scalability issue.

Another study by Agbehadji et al. (2021) [8] developed a clustering algorithm based on a nature-inspired approach for energy optimization in a heterogeneous wireless sensor network. Their approach focused on the heterogeneity of devices, energy requirements of nodes, and network characteristics. The performance of the proposed scheme was measured based on the number of data packets successfully received at the base station; data transmission and reception were also efficient in the network. In [8], performance was compared with existing models such as Distributed Energy-Efficient Clustering (DEEC), Developed Distributed Energy-Efficient Clustering (DDEEC), and Extended version of Distributed Energy-Efficient Clustering (EDEEC).

Jacob I. and Darney P. (2021) [9] proposed the Artificial Bee Colony (ABC) Optimization Algorithm as a method for enhancing routing in wireless networks. This algorithm is inspired by the foraging behavior of honeybees and is designed to optimize various performance metrics in wireless communication systems. The study aimed to provide a novel approach to routing that enhances efficiency and performance through the use of bio-inspired algorithms. Their algorithm initializes a population of artificial bees, which represent potential solutions (routing paths) in the network. Each bee is assigned a specific route to explore based on the network model. The behavior of the bees was simulated to mimic the natural foraging process. Each bee evaluates the quality of its assigned route based on performance metrics such as throughput, delay, and energy consumption. The bees communicate information about the quality of their routes to other bees, similar to how real bees share information about food sources. The bees assess the performance of their routes using predefined performance indicators. This evaluation helps determine which routes are more efficient and should be prioritized for data transmission. Based on the evaluations, the bees select the best routes to follow. The algorithm employs a mechanism to exploit the best-known routes while also exploring new paths to avoid local optima. If a bee finds a better route than its current one, it updates its position to this new route. They demonstrated that once the optimal paths are determined, data packets are transmitted along these routes. The algorithm ensures that packets are spread across multiple paths to enhance reliability and reduce the risk of packet loss due to interference or congestion. The proposed scheme is validated through simulations (e.g., using MATLAB), where various scenarios are tested to evaluate the performance improvements achieved by the ABC algorithm compared to existing routing protocols. While the proposed ABC optimization scheme presents a promising approach to enhancing routing in wireless networks, it lacks some capabilities, such as fast convergence rates, local optima, network scalability, and adaptability to changing network conditions. Addressing these limitations through further research and refinement could improve the algorithm’s robustness and applicability in diverse network scenarios.

Zhu et al. (2024) [11], in their study, proposed a Random Dual Strategy Artificial Bee Colony (RDABC) algorithm to address the challenges of low coverage accuracy and slow convergence speed in wireless sensor networks (WSNs). The RDABC focuses on enhancing the exploration and exploitation capabilities of the optimization process, thereby improving overall coverage and efficiency in WSNs. The RDABC randomly deploys sensor nodes within the designated field or geographical area for the coverage optimization task to begin. RDABC uses two search strategies: Classical Search Strategy and Elite Search Strategy. The update equation for the classical search, which was based on the original Artificial Bee Colony (ABC) algorithm, is computed in Equation (1) as:

(1) $$V_{t}=X_{t}+\emptyset (X_{t}-X_{k})$$

where $V_{t}$ is the new position of the bee, $X_{t}$ is the current position, $X_{k}$ is a randomly selected individual from the population, and ∅ is a random parameter that influences the search direction.

This strategy focuses on the best solutions found so far. The update equation for the elite search is computed in Equation (2) as:

(2) $$V_{t}={XE}_{t}+\emptyset (X_{best}-X_{k})$$

where *XE* is the central position of all elite solutions (top 10% of the population) and $X_{best}$ is the best individual in the population.

The RDABC incorporated a random selection method to switch between the classical and elite search strategies, allowing for flexible transitions and balancing exploration and exploitation. RDABC introduced a cross-mutation strategy derived from genetic algorithms. This strategy allows for multi-dimensional updates, which helps in exploring the solution space more effectively. The specific implementation details of the cross-mutation are not explicitly defined in the provided text, but it generally involves combining features from multiple solutions to create new candidate solutions. The RDABC iterates through the steps until a stopping criterion is met, such as a maximum number of iterations or a satisfactory level of coverage. The final output includes the optimized positions of the sensor nodes and the achieved coverage area. Although RDABC offers significant improvements over traditional methods, it may be faced with challenges like implementation complexity due to the dual search strategies and cross-mutation and being trapped in local optima, particularly in highly complex or multimodal optimization landscapes.

The Wolf Search Algorithm (WSA) operates based on the natural behaviors of wolves during hunting, incorporating principles of individual searching, memory utilization, and threat response [12]. WSA simulates the hunting behavior of wolves, where each wolf acts as an independent searching agent. The algorithm balances exploration (searching new areas) and exploitation (refining known good areas) to find optimal solutions.

Each wolf evaluates its current position and the positions of its peers within a defined visual range. The movement of a wolf is influenced by the quality of its position and the best position found by its peers; this is computed in Equation (3) as:

(3) $$x_{new}=x_{old}+\propto \cdot \left(x_{best}-x_{old}\right)+\beta \cdot escape()$$

where $x_{new}$ is the new position of the wolf, $x_{old}$ is the current position of the wolf.

$x_{best}$ is the best position found by the wolf or its peers, ∝ is a coefficient that controls the attraction towards the best position, β is a coefficient that controls the randomness of the escape movement, escape() is a function that generates a random position to jump to, simulating the wolf’s response to threats.

When a wolf encounters a threat (simulated as a random event), it jumps away from its current position to avoid being trapped in local optima. This is akin to a mutation in genetic algorithms. The escape movement is defined in Equation (4) as:

(4) $$x_{scape}=x_{old}+random(-d,d)$$

where *d* is a predefined distance that determines how far the wolf can jump away from its current position, random (−*d*, *d*) generates a random value within the range of −*d* to *d*.

WSA incorporates a memory mechanism where wolves remember previously visited positions. This memory helps prevent revisiting the same positions and enhances search efficiency. Each wolf evaluates the quality of its current position based on a fitness function, which is problem-specific. The fitness function determines how close the current position is to the optimal solution. The fitness function *f*(*x*) is defined in Equation (5) as:

(5) $$f\left(x\right)=Objective\;Function(x)$$

The WSA follows the general steps outlined below:

**Step 1:** Initialize a population of wolves with random positions.

**Step 2:** Evaluate the fitness of each wolf’s position.

**Step 3:** For each wolf, determine the best position found by itself and its peers.

**Step 4:** Update the position of each wolf using the movement formula.

**Step 5:** If a threat is encountered, apply the escape movement.

**Step 6:** Repeat the evaluation and updating process for a set number of iterations or until convergence criteria are met.

Rami Reddy et al. (2023) [13] proposed a scheme based on the improved Grey Wolf Optimization (EECHIGWO) algorithm to enhance energy efficiency, average throughput, network stability, and overall network lifetime in wireless sensor networks (WSNs) through optimal selection of cluster heads (CHs). The EECHIGWO specifically focused on addressing the challenges associated with traditional clustering methods, such as premature convergence, lack of population diversity, and the imbalance between exploration and exploitation in the optimization process. The EECHIGWO incorporated elements of the wolf search algorithm (WSA) to improve the efficiency and effectiveness of cluster head selection in WSNs, leading to enhanced network performance and longevity. The proposed EECHIGWO defines a fitness function that evaluates the potential CHs based on criteria such as residual energy, distance to the base station, and intra-cluster distance. The WSA uses this fitness function to guide the selection process, ensuring that the chosen CHs are optimal for energy efficiency and network stability. The EECHIGWO algorithm demonstrated a substantial improvement in network lifetime, with reported enhancements of up to 333.51% compared to several existing protocols, such as SSMOECHS, FGWSTERP, and LEACH-PRO. Although the EECHIGWO aims to balance exploration and exploitation, there may still be scenarios where it could get trapped in local optima, especially in highly complex or non-linear search spaces. The EECHIGWO may also introduce additional complexity in terms of implementation and parameter tuning.

Jiang et al. (2016) [14] developed an underwater sensor network redeployment algorithm based on the wolf search algorithm (RAWS) to optimize how sensors are deployed in underwater environments. The focus of RAWS was to address challenges like inefficient network coverage, high computational complexity, energy inefficiency, obstacles, and environmental changes affecting underwater sensor networks’ performance. The RAWS allows each node to perform an independent search for monitoring purposes. This does not enable sensors to exchange information with one another, which reduces communication overhead and computational complexity. Sensors move towards targets when detected or explore freely when no targets are present. The proposed RAWS included an escape mechanism that allows sensors to change their direction randomly when they encounter an obstacle to avoid it. This feature helps prevent sensor node failure and allows the algorithm to escape local optima during the search process. The node coverage for each node $s_{i}$, which was computed in Equation (6) as:

(6) $$N_{event}\left(s_{i}\right)=\sum_{1}^{n}f\left(p_{j},s_{i}\right)\neq 0$$

where, $f\left(p_{j},s_{i}\right)$ is a function that determines if the target $p_{j}$ is within the sensing range of the node $s_{i}$.

The performance of the proposed RAWS was evaluated through simulations. The RAWS results demonstrated improvements in network coverage, energy conservation, and obstacle avoidance compared to other existing algorithms, such as the artificial fish swarm algorithm. Although the proposed RAWS has demonstrated some improvement in network performance, it may be sensitive to the initial random deployment of nodes. If nodes are poorly positioned initially, it may take longer for the algorithm to achieve optimal coverage, or it may converge to a suboptimal solution. Also, the escape mechanism employed by RAWS relies on the ability of nodes to detect obstacles. If the detection range is limited or if obstacles are not accurately represented in the model, nodes may fail to avoid them effectively, leading to potential collisions or failures.

## 3. Theoretical Framework

### 3.1. Bee Colony Optimization Algorithm

The Bee Colony Optimization (BCO) algorithm is a nature-inspired optimization scheme based on the foraging behavior of honeybee colonies [15]. BCO imitates the food-foraging behavior of honeybees to find solutions to complex optimization problems. Honeybee colonies show incredible efficiency in searching for food sources in their immediate environment and embark on complex foraging behavior to locate, collect, and bring food (nectar) to the hive [13].

The honeybee communicates via dance movements and performs a distinctive “waggle dance”, signaling or sending information about the location of food sources to other bees in the colony [5]. Honeybees share details like the distance, direction, and quality of food sources through these dance movements, letting other bees optimize their foraging routes and efficiently gather nectar. Honeybee colonies collectively work to gather nectar from multiple sources of food [6]. The collaborative activities of individual honeybees contribute to the overall success of the colony in getting enough food for survival and growth.

Like honeybee colonies, the BCO algorithm focuses on an efficient search for high-quality solutions by coordinating the search behavior of artificial bees [6]. The BCO algorithm’s iterative nature and adaptive search technique make it a strong optimization strategy for solving complex optimization problems. The BCO algorithm comprises two main phases, the forward pass and the backward pass, which can be described as follows:

#### 3.1.1. Forward Pass

At this stage, every honeybee finds a solution by exploring the search space around its colony. The quality of the solution found by a honeybee affects the probability of that honeybee’s continuous independent search or following another honeybee [5]. The forward pass can be expressed in Equation (7) as:

1. Probability of Selecting a Neighbor Solution:

(7) $$P_{ij}=\frac{f(S_{ij})}{\sum_{k=1}^{n}f(S_{ik})}$$

where, $P_{ij}$ is the probability that a honeybee ‘*i*’ selects a neighbor solution $S_{ij}$ during the exploration phase. *f*() evaluates the quality of a solution. Thus, a lower fitness value shows a better solution to minimize a problem. *f*($S_{ij}$) calculates the fitness of the neighbor solution under consideration. $\sum_{k=1}^{n}f(S_{ik})$ is the sum of fitness values of all neighbor solutions available to honeybee *i*. Dividing the fitness of the current neighbor solution by the sum of the fitness values of all neighbors determines the probability of a honeybee *i* choosing a solution $S_{ij}$ for further exploration.

From Equation (4), each honeybee examines the quality of neighbor solutions based on a fitness function *f*. $S_{ij}$ is the probability of selecting a neighbor solution, which is computed as the ratio of the fitness of the neighbor solution to the sum of the fitness values of all neighbor solutions. This probability determines the likelihood of a honeybee choosing a particular neighbor solution during a search [15].

2. Local Search Equation

After selecting a neighbor solution, the honeybee performs a local search around that solution, which can be expressed in Equation (8) as follows:

(8) $$S_{ij}=S_{ij}+\emptyset_{ij}\times \left(S_{ij}-S_{kj}\right)$$

where $S_{ij}$ is the new solution, updated by moving toward the neighbor solution $S_{kj}$ which is based on a random factor $\emptyset_{ij}$. This local search method allows honeybees to scout the search environment more effectively and potentially discover better solutions [15].

This study introduces the Wolves Search Algorithm (WSA) into the local search operation of honeybees as the coefficient vector. The coefficient vector represents a set of parameters that influences the movement and decision-making processes of the wolves during the search for optimal solutions [16]. Again, it allows each wolf to balance their search in new solution space and then refine the search around known good solutions. This ensures the adaptability of search in non-explored areas. The coefficient vector ***A*** is calculated based on factor **a**, which decreases linearly from 2 to 0 throughout iterations [17]. This implies that ***A*** changes its values at each time step, influencing the wolves’ movement towards the prey. Hence, given Equation (9):

(9) $$A=2\cdot a\cdot r_{1}-a$$

where **a** is a linearly decreasing parameter from 2 to 0 over the iterations, $r_{1}$ is a random vector in the range [0, 1]. Therefore, the honeybees’ local search formula is now computed in Equation (10) as:

(10) $$S_{ij(new)}=S_{ij(current)}+A_{ij}\times \left(S_{ij(best)}-S_{kj(current)}\right)$$

where, $S_{ij(current)}$ is the current value of *j* in the solution $S_{i}$. $S_{ij(best)}$ is the corresponding *j* value from the best solution $S_{k}$ in the population. $A_{ij}$ is the coefficient vector derived from the WSA, which replaces the random factor $\emptyset_{ij}$. The ability of WSA to evaluate positions based on the quality of solutions found by peers makes it suitable for optimizing routing paths and clustering in WSNs, where finding optimal solutions is crucial for performance and energy efficiency. The combination of the Bee Colony Optimization (BCO) algorithm with the Wave Search Algorithm (WSA) can enhance the performance of optimization tasks in Wireless Sensor Networks (WSNs) as follows:

i. The BCO algorithm is known for its effective exploration capabilities due to its nature-inspired approach based on the foraging behavior of honeybees. However, it can sometimes suffer from premature convergence to suboptimal solutions. The WSA, on the other hand, is designed to explore the search space more thoroughly by simulating wave propagation. By combining these two algorithms, the strengths of both can be leveraged, leading to improved exploration and exploitation of the solution space.
ii. The integration of WSA can help accelerate the convergence of the optimization process. While BCO may take longer to find optimal solutions due to its reliance on the collective behavior of bees, the wave propagation mechanism in WSA can guide the search process more efficiently, allowing for faster identification of high-quality solutions.
iii. One of the challenges in optimization problems is the tendency of algorithms to get trapped in local optima. The combination of BCO and WSA can enhance robustness against this issue. The wave search mechanism can introduce diversity in the search process, helping to escape local optima and explore new areas of the solution space.
iv. In WSNs, the environment can be dynamic, with changing conditions and requirements. The hybrid approach can provide greater adaptability, as the wave search can quickly adjust to changes in the search landscape, while BCO can maintain a balance between exploration and exploitation.
v. In WSNs application, energy efficiency is critical; combining BCO with WSA can lead to more effective resource management. The hybrid algorithm can optimize the selection of cluster heads and data transmission paths, ensuring that energy consumption is minimized while maintaining high levels of data delivery and network performance.
vi. The combination of BCO and WSA can be applied to a wide range of optimization problems beyond WSNs, including routing, scheduling, and resource allocation in various fields. This versatility makes the hybrid approach a valuable tool for researchers and practitioners looking to solve complex optimization challenges.

#### 3.1.2. Backward Pass

Bees return to the hive after exploring the search space and comparing their solutions to further adjust their search strategies to avoid getting trapped in local optima. Thus, the Backward Pass Equations are described as:

1. Comparison of Solutions

The comparison solution can be expressed in Equation (11) as:

(11) $$Compare\left(S_{ij},S_{kj}\right)=\left\{\begin{array}{l} 1,\;if\;f\left(S_{ij}\right)<f\left(S_{jk}\right)\\ 0,\;otherwise \end{array}\right.$$

The backward pass compares the current solution $S_{ij}$ found by honeybees with the neighbor solution $S_{jk}$ concerning their fitness values. If the fitness of the neighbor solution is good (lower in the case of minimization problems), the honeybee updates its solution to move towards a better solution. The comparison process helps honeybees adjust their search direction based on the quality of solutions found by other bees.

2. Update Solution

The update strategy is defined in Equation (12) as:

(12) $$S_{ij}=S_{ij}+∆S_{ij}\times Compare\left(S_{ij},S_{kj}\right)$$

where the Honeybee updates its solution $S_{ij}$ by integrating a change $∆S_{ij}$ based on the comparison result. If the neighbor’s solution $S_{kj}$ is better, the honeybee adjusts its solution toward the neighbor’s solution. This update mechanism allows bees to learn from the collective search behavior and improve their solutions iteratively.

### 3.2. BCO Algorithm Workflow

**Step 1:** Bees are initialized with random solutions in the search space.

**Step 2:** Bees probabilistically explore the search space, select neighbor solutions, and perform a local search. This is known as the Forward Pass.

**Step 3:** Bees perform a Backward Pass. Thus, bees compare their solutions with neighbors, update their solutions based on comparison results, and share information.

**Step 4:** The forward and backward passes are repeated a specified number of times or until a stopping criterion is met.

**Step 5:** To determine their quality, solutions are evaluated based on an objective function.

**Step 6:** The algorithm aims at converging towards optimal solutions by repeatedly improving the solutions through exploration and exploitation.

### 3.3. K-Means Algorithm

The K-means algorithm is a clustering method in the field of data mining and machine learning [18]. The K-means algorithm was introduced by Stuart Lloyd in 1957 as a technique for pulse-code modulation (PCM) quantization in signal processing [18,19]. It was later rediscovered by MacQueen, who, in 1967, introduced the algorithm as a clustering technique in the context of pattern recognition. The K-means algorithm was initially applied in signal processing and image compression, which have proven to be very effective. It is also known for its simplicity, efficiency, and effectiveness in partitioning data into clusters based on similarity [20].

Integrating the K-means algorithm into the Bee Colony Optimization (BCO)) helped to solve complex optimization problems that required clustering [21,22]. The K-means clustering method used an iterative procedure to assign data points to clusters based on centroid proximity and update centroids until convergence. Although the K-mean is effective for many applications, it has some limitations relating to initialization, cluster shape assumptions, and scalability [2].

The K-Means clustering algorithm or scheme is a well-known partitioning technique that aims to divide *n* data points into *K* clusters. The scheme repeatedly assigns data points to the nearest cluster centroid (cluster heads) and updates the centroids based on the mean of the data points in each cluster.

The following steps constitute the process involved in K-Means clustering:

**Step 1: Initialization**

- Select the number of clusters, *K*.
- Randomly initialize *K* cluster centroids, $C=\left\{c_{1},c_{2},\ldots ,c_{K}\right\}$.

**Step 2: Assignment**

- For every data point $x_{i}$, calculate the distance to each centroid $c_{j}$ using a distance metric (e.g., Euclidean distance).
- Assign the data point $x_{i}$ to the cluster with the nearest centroid: ${argmin}_{j}=\left\{{\left\|x_{i}-c_{j}\right\|}^{2}\right\}$.

**Step 3: Update**

- Update the cluster centroids by calculating the mean of all data points assigned to each cluster: $c_{j}=\frac{1}{\left|S_{j}\right|}\sum_{x_{i}\in S_{j}}x_{i}$ where $S_{j}$ is the set of data points assigned to cluster *j*.

**Step 4: Convergence**

- Iterate the Assignment and Update steps until convergence criteria are met. Convergence can be based on the change in cluster assignments or centroid positions.

**Step 5: Final Result**

- The algorithm converges when the cluster assignments stabilize and the centroids no longer change significantly. The final result is a set of *K* clusters with their respective centroids.

K-Means clustering technique aims to minimize the within-cluster sum of squares, where the objective function is: $J=\sum_{i=1}^{K}\sum_{x\in S_{i}}{\left\|x-c_{i}\right\|}^{2}$ where $S_{i}$ is the set of data points assigned to cluster *i* and $c_{i}$ is the centroid of cluster *i*. Updating cluster assignments and centroids repeatedly, K-Means seeks to find a partition of the data that minimizes the total within-cluster variance. The iterative nature of the K-mean algorithm allows for efficient assignment and updating of clusters, making it effective for organizing nodes in WSNs. However, K-means has limitations, such as sensitivity to initialization and assumptions about cluster shapes. Therefore, by integrating K-means with BCO, the study will leverage its strengths while addressing its weaknesses, particularly in terms of energy consumption and network lifetime.

## 4. Proposed Clustering Technique in Wireless Sensor Networks (WSNs)

The proposed clustering technique combines the forward and backward passes repeatedly to generate the optimal solutions. In this regard, the sensor nodes (SN) perform the forward and backward passes repeatedly for a specified number of times to locate a cluster head (CH) with the optimal distance. One advantage of the proposed clustering technique is its ability to search all available nodes and then optimize energy in transmitting data packets between sensor nodes (SN) and cluster heads (CH) and among CHs. Optimizing the energy takes into consideration the distance to communicate in routing data packets to the sink node or base station through another CH.

The proposed scheme combines WSA, K-means, and Bee Colony Optimization to optimize the performance of WSNs. The integration of K-means with BCO (K-BCO) aims to enhance the clustering process, improve energy efficiency, and extend the network’s operational lifetime. The combination of WSA, K-means, and BCO is because there is a need to develop a robust and efficient clustering algorithm that addresses the challenges of energy consumption and network performance in WSNs. This integrated approach aims to provide more stable and optimal solutions, ultimately leading to improved network lifetime and efficiency.

### 4.1. Cluster Formation and Cluster Head Selection

Clustering enhances routing in WSNs by improving energy efficiency, facilitating data aggregation, and supporting the development of efficient routing protocols tailored for hierarchical network structures [1,8]. This routing technique models the food search method by honey bees, where SNs find the optimal distance to a CH. CHs also use optimal distance to communicate and route data packets to the sink node or base station through another CH. In this regard, the forward and backward passes are performed iteratively to locate a CH with the optimal distance. The following subsections describe the cluster formation and bit error determination stages.

During this process, the energy model and Bit Error Rate (BER) determination are considered in cluster formation and CH selection at every given time. These are described as follows:

- *Energy model*

The study adopts the energy model by [10] to determine the energy requirement for a sensor or CH to send *n-bit* of a data packet over a distance *d*. Hence, the transmission energy is defined in Equation (13) as:

(13) $$E_{tx}\left(n,d\right)=E_{xtelec}\left(m\right)+E_{txampt}(n,d)$$

where $E_{xtelec}\left(m\right)$ represents the energy consumed for electronic transmission and $E_{txampt}(n,d)$ is the energy consumed for amplification during transmission. Hence, the energy consumption *f*(*x*) is expressed in Equation (14) as:

(14) $$f\left(x\right)=\left\{\begin{array}{c} n\times E_{elec}+(n\times E_{fs}\times d^{2}),\;d<d_{0}\\ n\times E_{elec}+(n\times E_{amp}\times d^{4}),\;d\geq d_{0} \end{array}\right.$$

where $f\left(x\right)$ is the energy consumption for sending a message over a certain distance in the network. Where $E_{elec}$ represents the energy consumed for the electronic transmission, $E_{fs}$ represents the energy required for signal amplification, d is the distance over which the message is being transmitted and $E_{amp}$ represents the energy consumed for signal amplification. Furthermore, the energy consumed for receiving the message from a sender is computed in Equation (15) as:

(15) $$E_{rx}\left(n\right)=n\times E_{elec}$$

The residual energy ($E_{res}$) of a sensor node is calculated using Equation (16):

(16) $$E_{res}=E_{in}-E_{tx}\left(n,d\right)+E_{rx}(n)$$

where, $E_{in}$ is the initial energy of the sensor node, $E_{tx}\left(n,d\right)\;$ is the transmission energy calculated earlier, and $E_{rx}(n)$ is the energy consumed for receiving the message.

The study utilized the Euclidean distance formula to compute the distance *d* between SN and CH and between CHs and the sink node or the base station (BS). The Euclidean distance formula is a fundamental concept in mathematics that calculates the straight-line distance between two points in Euclidean space [23,24]. In a two-dimensional space (such as a Cartesian plane), the Euclidean distance *d* between two points $\left(x_{1},y_{1}\right)$ and $\left(x_{2},y_{2}\right)$ is expressed in Equation (17):

(17) $$d=\sqrt{{\left(x_{2}-x_{1}\right)}^{2}+{\left(y_{2}-y_{1}\right)}^{2}}$$

1. Bit Error Rate (BER) Determination

The Bit Error Rate (BER) is a measure of the percentage or fraction of bits in a data stream that has been corrupted during transmission [25,26]. It represents the ratio of the number of erroneous bits received to the total number of bits transmitted over a specific period, which is expressed in Equation (18).

(18) $$BER=\frac{B_{tner}}{B_{tnt}}$$

where $B_{tnt}$ represents the total number of bits transmitted and $B_{tner}$ is the total number of erroneous bits received.

A lower BER value indicates a higher level of data accuracy and reliability in the communication system. For instance, a sensor of BER of 0.01 means that 1 out of every 100 bits transmitted is received in error. BER is crucial in assessing the quality of communication channels, especially in scenarios where noise, interference, or channel impairments can introduce errors in the transmitted data.

The optimal BER for wireless communication systems is typically set based on the required quality of service (QoS) for the application [25]. In high-reliability applications such as medical devices or critical infrastructure monitoring, a very low BER of $10^{-9}$ may be considered as optimal. The BER requirements of $10^{-12}\;\mathrm{to}\;10^{-15}$ may be considered optimal in optical systems to ensure error-free transmission over long distances.

### 4.2. Proposed K-BCO Algorithm

The proposed algorithm consists of several steps, which are outlined in Algorithm 1:

**Algorithm 1:** K-BCO***Start:*** **Step 1:** Set the parameters for CH selection and CH search **Step 2:** Determine the number of clusters (k-means) **Step 3:** Select CHs    Start loop    Compute energy levels (Equation (13)) and (Equation (16))    Evaluate energy consumption (Equation (12))    Computer BER (Equation (18))      If $f\left(x\right)\;is\;low\;and\;BER\leq 10^{-12}$        Select CH       End if          End loop **Step 4:** SN search for CH (Equation (9))    Start loop       If d≤12.5 m,        Connect to CH     else       Search for new CH (Equation (7))     End if       End loop **Step 5:** count the number of sensors in a Cluster    Start loop     If count=5,      Stop sensor acceptance     End if        End loop **Step 6:** Establish Communication **End**

### 4.3. Flowchart of the Proposed K-BCO Algorithm

**Figure 1:**
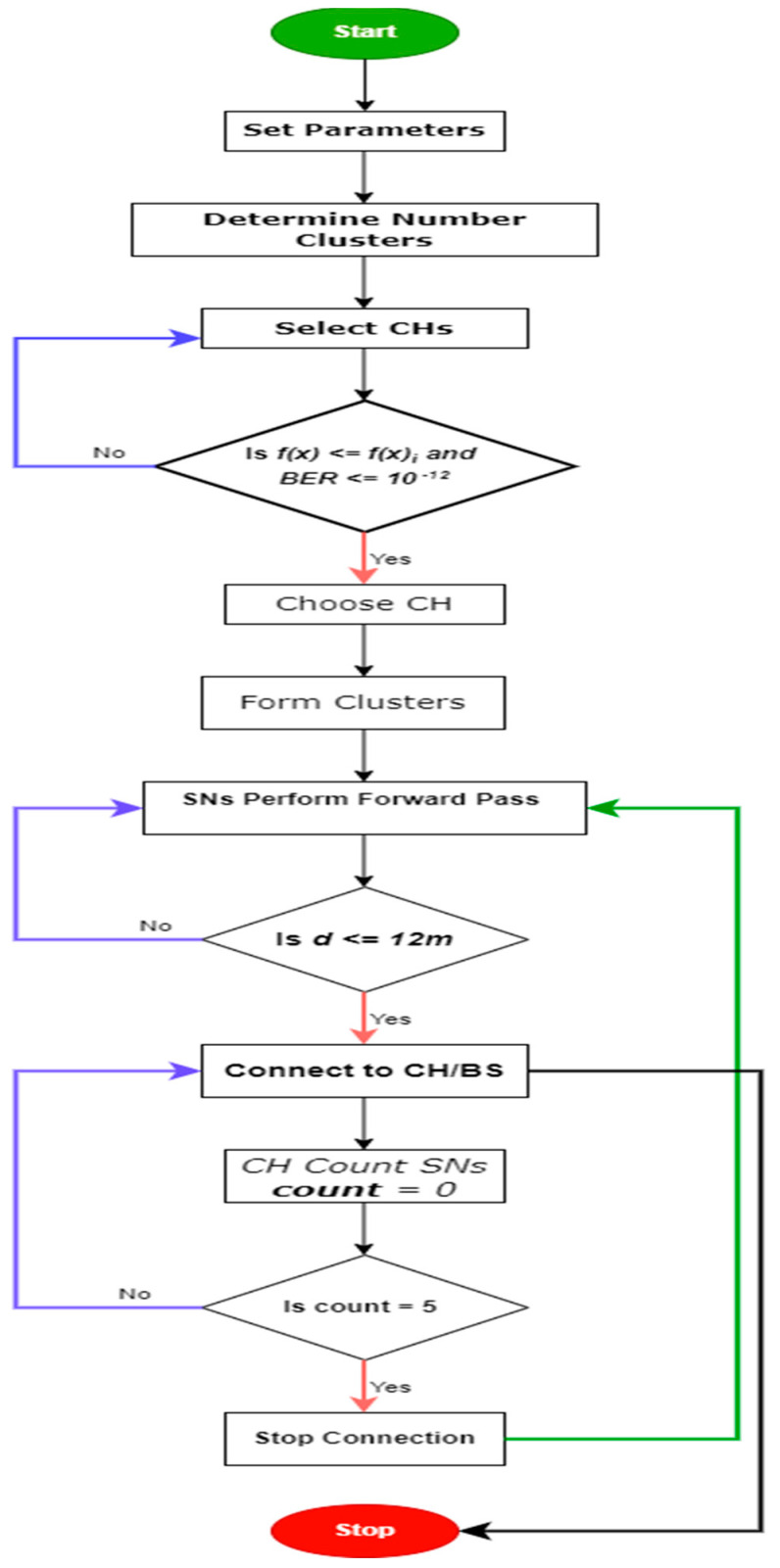
The Flowchart of the Proposed K-BCO Algorithm.

From Figure 1, in implementing the proposed algorithm, the algorithm first sets the parameters for energy level, BER, distance, cluster head selection, and cluster head search. The scheme then determines the number of clusters depending on the number of sensors deployed using k-mean.

Secondly, the algorithm selects CHs based on the parameters for the CH selection. Afterward, the sensor within a cluster searches and connects to the closest CH using the forward pass technique based on the distance between the sensor and the CH. The proposed algorithm allows a maximum of five (5) sensors to be connected to a CH at each time. This ensures that mobile sensors do not overburden any CH within the network.

### 4.4. Computational Complexity of the Proposed K-BCO

We present the complexity of the proposed K-BCO in terms of big O complexity in Algorithm 2, although this study focuses on error rate, data delivery rate, and execution time.


**Algorithm 2: Computational complexity**
**Step 1:** Set the parameters → O(1) **Step 2:** Determine the number of clusters → $O(m^{2}\cdot r\cdot d)$   • *m* is the number of sensors (data points).   • *r* is the number of iterations required for convergence. **Step 3:** Select CHs → O(m) **Step 4:** SN search for CH → O(m·k)  Where $k=\frac{m}{5}$, 5 is the number of sensors per cluster **Step 5:** count the number of sensors in a Cluster → O(m) **Step 6:** Establish Communication → O(1)


## 5. Experimental Settings of K-BCO in WSN

Simulation of the proposed algorithm was achieved using Python version 3.12 and PyCham version 241.18 on the Windows 11 64-bit operating system. Python is a component-based simulation environment that supports the simulation of various communication networks, including wireless sensor networks [27]. It provides a flexible framework for modeling network protocols and scenarios.

The proposed scheme considers about 250 wireless sensor nodes distributed over a field or geographical area of $400\;m^{2}$ randomly. Clusters are formed, and cluster heads (CHs) are selected based on their energy efficiency and transmission error rates (Bit Error Rate (BER)). Sensors locate the CHs using the optimal distance. The study also considers only five sensors as a cluster around each CH at any given time. Table 1 below shows the experimental settings.

A random dataset on humidity and temperature was generated for each sensor within the network using the uniform distribution function in Python. A uniform distribution function is a type of probability distribution in which all outcomes are equally likely to occur [28,29]. Thus, every value within a specified range has the same probability of occurring. To generate data for randomly distributed sensors in a field using a uniform distribution, the following steps were followed:

Step 1: Define the Field Dimensions

Let the dimensions of the rectangular field be defined as:


$$\begin{array}{c} \mathrm{Width}\;W\;=\;400\;m\\ \mathrm{Height}\;H\;=\;400\;m \end{array}$$

Step 2: Specify the Number of Sensors

Let *N* be the number of temperature sensors to be deployed:


N = 250

Step 3: Set Constraints

Define the minimum distance *d* that must be maintained between any two sensors:


d = 12 m

This is to avoid long distances between sensor nodes and CHs. Long distances between sensor nodes and CHs may lead to high energy consumption during data transmission from sensor nodes to CHs. Therefore, this limits the lifespan and efficiency of the network.

Step 4: Generate Random Coordinates

Let $\left(x_{i},y_{i}\right)$ represent the coordinates of the *i*-th sensor, where *i* = 1, 2, …, *n*. The position coordinates of sensors are generated using Equation (19):

(19) $$\left(x_{i},y_{i}\right)=\left(W\cdot u_{i},H\cdot v_{i}\right)$$

where $u_{i}$ and $v_{i}$ are independent random variables uniformly distributed between 0 and 1. Thus,

$$x_{i}=W\cdot u_{i}$$

(20) $$y_{i}=H\cdot v_{i}$$

Therefore, humidity *h* and temperature *t* are generated for each sensor within the network using Equation (18):

$$h_{i}=100\cdot \;q_{i}$$

(21) $$t_{i}=40\cdot \;q_{i}$$

After generating the above data for each randomly distributed sensor, the data was sent to the sink node through the respective CHs of each cluster.

The algorithm to implement random distribution is simplified as shown in Algorithm 3:

**Algorithm 3:** Simplified Random Distribution Algorithm**Step 1:** initialize W, H, N, $u_{i}$, $v_{i}$, $q_{i}$ **Step 2:** set *d* **Step 3:** Generate random coordinates **Step 4:** Output random distribution value

## 6. Simulation Results of the Proposed K-BCO Algorithm

**Figure 2:**
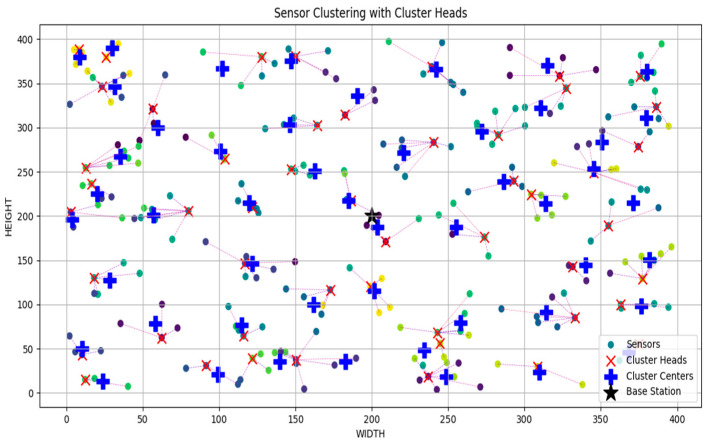
Simulation of sensors, cluster heads, cluster centers and base stations within the 400-m square area.

Figure 2 shows the clustering performed on 250 sensors in a 400-square-meter geographical area.

Figure 2 shows the simulation results of heterogeneous sensors, cluster heads, cluster centers and base station (BS) within the 400-m square field using Python. It is observed that the sensors within the network were randomly distributed on the field and grouped in clusters. Cluster heads (CHs) were selected for each cluster where the sensor is connected to a CH. Each CH is connected to at most five sensors depending on the distance between the CH and the sensor and the number of sensors already connected to the CH. It is also observed that the position of the BS was at the center of the field. Cluster centers were also indicated, but that does not determine the position of the CH since the selection of the CHs was based on different criteria.

### 6.1. Performance Evaluation of K-BCO Algorithm

The study evaluated the performance of the proposed K-BCO based on average error rate (AER), average data delivery rate (ADDR), and average energy consumption (AEC) for a data packet to be transmitted from heterogeneous sensors to their respective cluster heads. Also, the K-BCO was assessed in terms of execution time (ET) in Python. K-BCO was executed 50 times, and the various matrix values were taken. Table 2 shows the performance results in terms of AER, ADDR, AEC and ET of the K-BCO.

**Figure 3:**
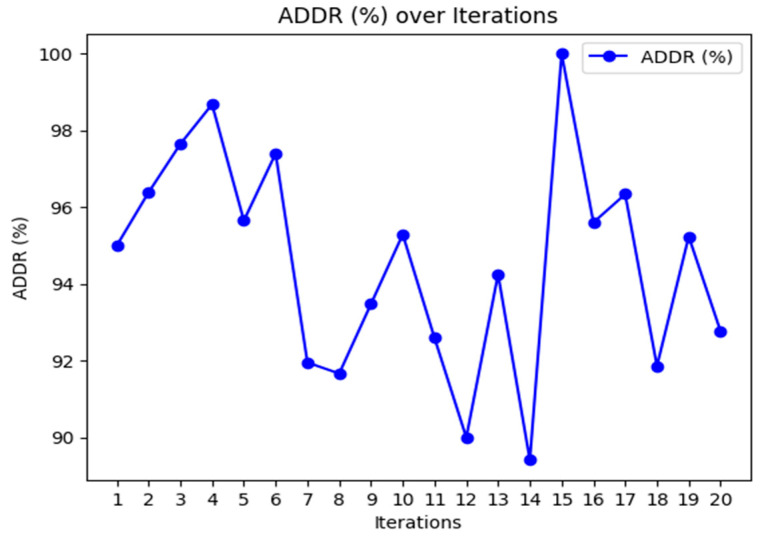
Data delivery rates of K-BCO over 20 iterations.

**Figure 4:**
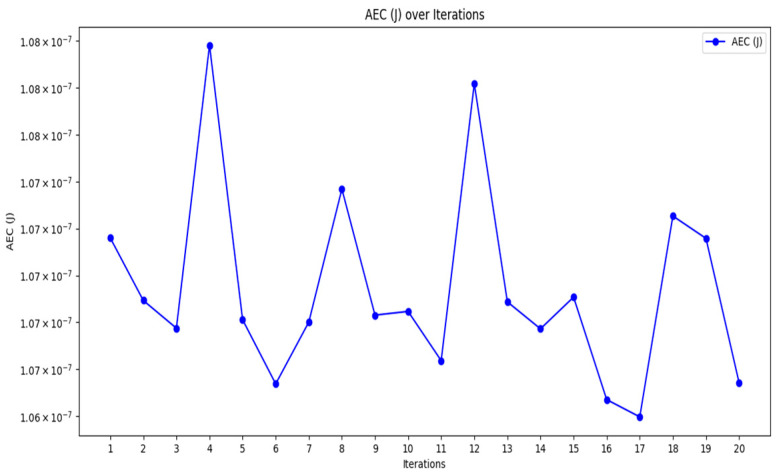
Energy consumption of K-BCO over 20 iterations.

**Figure 5:**
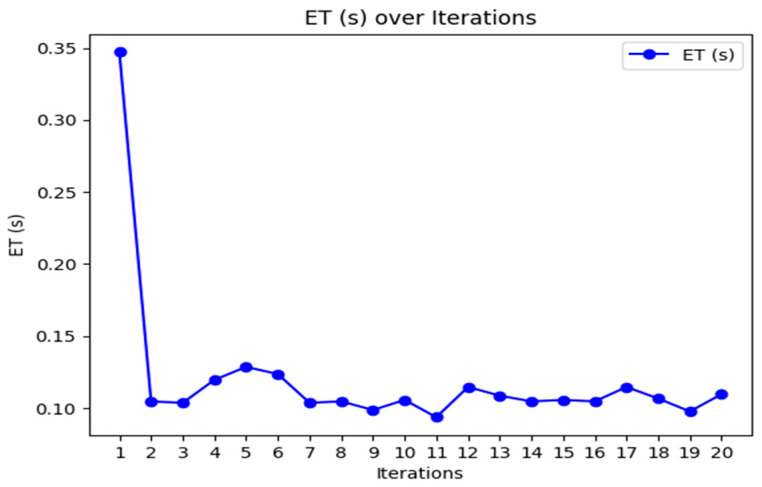
Average execution time of K-BCO over 20 iterations.

Figure 3, Figure 4 and Figure 5 provide the graphical representation of the performance results of the proposed K-BCO in terms of data delivery rate, energy consumption values, and execution time for the first 20 iterations.

Figure 3, Figure 4 and Figure 5 show the performance of the proposed K-BCO regarding average data delivery rate (ADDR), execution time (ET), and average energy consumption (AEC) or energy cost for a data packet to be transmitted from heterogeneous sensors to their respective cluster heads. Figure 3 illustrates the proposed ADDR, whilst Figure 4 and Figure 5 show AEC and ET, respectively. The ADDR was computed based on percentage (%). The ADDR ranges from 92% to 100% of data delivered successfully to CHs. AEC and ET were computed in joules (J) and seconds (S), respectively. The performance of the K-BCO in terms of AEC and ET for the first 20 iterations ranges from 1.064 × 10^−7^ J to 1.079 × 10^−7^ J and 0.09 s to 0.35 s, respectively. From Table 2, the average error rate (AER) ranges from 0.0% to 10.0% of data that were lost or corrupted during transmission from sensors to the CHs.

### 6.2. Comparison of the Proposed K-BCO

The performance of the algorithm was compared with H-LEACH, Distance Based Clustering Protocol (DBCP) and ABC-ACO by [10,30,31], respectively, using the same parameters like the number of sensors, clusters, data set, and the network area size over 50 iterations. Table 3 also measured the performance of the proposed clustering algorithm in terms of the execution time.

**Figure 6:**
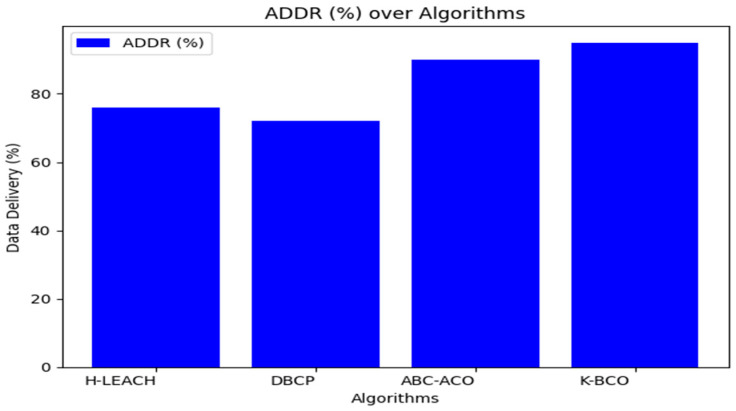
Average data delivery rates of the proposed K-BCO comparison with H-LEACH, DBCP and ABC-ACO.

**Figure 7:**
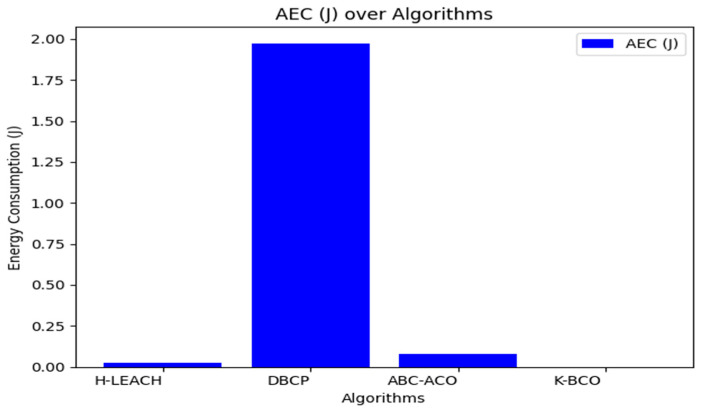
Average energy cost of the proposed K-BCO comparison with H-LEACH, DBCP and ABC-ACO.

**Figure 8:**
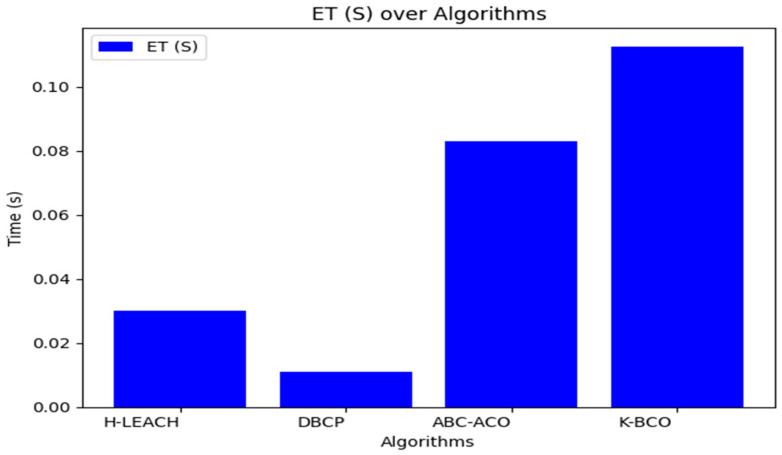
Execution time of the proposed K-BCO comparison with H-LEACH, DBCP and ABC-ACO.

From Table 3, the proposed clustering algorithm performed better than H-LEACH, DBCP, and ABC-ACO in terms of energy consumption by sensors for sending data to the CHs. Figure 6, Figure 7 and Figure 8 show a graphical representation of the performance of the proposed K-BCO against H-LEACH, DBCP and ABC-ACO.

Figure 6, Figure 7 and Figure 8 show a comparison of the proposed K-BCO against H-LEACH, DBCP, and ABC-ACO based on average data delivery rate (ADDR), terms of execution time (ET), and average energy consumption (AEC) or energy cost for a data packet to be transmitted from sensors to their respective cluster heads for ten iterations. The average of the results obtained from the 50 iterations for the various algorithms was evaluated, as shown in Table 3. From the figures, the K-BCO is a better candidate in terms of error resistance, data delivery in WSN, and energy consumption than H-LEACH, DBCP, and ABC-ACO.

## 7. Discussion of Results

The proposed method was simulated in a heterogenous WSN environment where sensor nodes were randomly distributed with varying. The heterogeneity in node mobility and positioning requires a flexible clustering approach that can adapt to changes in the network topology. The performance of our proposed algorithm was measured in terms of algorithm execution time, error rate, delivery rate, and energy consumption rate when packets of data are transmitted from the sensor nodes to the CHs. The execution time is the total time taken to complete a specific computational task or simulation [32]. The error rate is a measure of the number of packets that are lost or corrupted during transmission as compared to the total number of packets sent. The delivery rate, also known as the packet delivery ratio (PDR), is a measure of the effectiveness of the network in successfully delivering packets from the source to the destination (e.g., from sensor nodes to the sink) [33]. It is the ratio of the number of packets successfully received by the destination, in this case, the base station, to the total number of packets sent from the sensor node. Energy cost is the total energy expense incurred by sensors for generating and transmitting data to their respective CHs [34].

The Average Energy Cost was computed as the average energy consumed by each sensor when transmitting to its respective cluster head. The average error rate is the average error that occurs across all sensors when transmitting to their respective cluster heads, whilst the average delivery rate is the average of successfully delivered data from all sensors to their respective CHs within the network.

From Table 3, the proposed K-BCO performs better than H-LEACH, DBCP and ABC-ACO in terms of average error rate (AER), average data delivery rate (ADDR), and average energy consumption (AEC) or energy cost for a data packet to be transmitted from sensors to their respective cluster heads. The K-BCO also shows 20.15%, 24.92% and 5.18% success in data delivery over H-LEACH, DBCP and ABC-ACO, respectively. In terms of energy consumption by sensors, the K-BCO shows lesser energy usage than H-LEACH, DBCP, and ABC-ACO. However, the limitation of the proposed K-BCO is that it did not perform well in terms of execution time (ET) compared to all three state-of-the-art algorithms.

## 8. Conclusions

The study presents a significant improvement in the field of Wireless Sensor Networks (WSNs) through the development of the K-BCO algorithm for clustering. By integrating BCO with WSA, the proposed clustering algorithm effectively addresses the challenge of energy consumption during data transmission in WSNs. The combination of the BCO with WSA enhanced the performance, robustness, and adaptability of the resulting hybrid approach. This combination led to more efficient solutions in dynamic and resource-constrained environments like Wireless Sensor Networks. The results from extensive simulations demonstrate that the K-BCO not only improves the reliability and efficiency of cluster formation but also optimizes energy consumption, thereby prolonging the network’s lifetime. The heterogeneity of sensors in the study is a critical factor that influences the design and implementation of the K-BCO algorithm by addressing the challenges posed by diverse sensor capabilities, energy levels, data generation rates, dynamic topologies, and application requirements.

The findings indicate that the proposed approach leads to a notable enhancement in the performance metrics of WSNs, including reduced error rates, energy cost or energy consumption and improved packet delivery ratios. Thus, although the K-BCO performed highly in terms of packet delivery, it consumed less energy, leading to a prolonged lifespan for the entire network. This study underscores the potential of leveraging collective behaviors observed in nature to solve complex problems in network optimization.

Future work should focus on further refining the algorithm and exploring its applicability in various real-world scenarios, including dynamic environments and heterogeneous sensor networks. Again, future work should focus on addressing the execution time of the proposed K-BCO. Overall, the proposed K-BCO framework offers promising implications for the deployment and operation of WSNs, paving the way for more efficient and sustainable wireless communication systems.

## Figures and Tables

**Table 1:** Experimental settings.

Software	Operating System	Area Size	Number of Sensors
Python version 3.12 PyCham version 241.18	Windows 11 64-bit	$400\;m^{2}$	250

**Table 2:** Performance results of K-BCO.

Iterations	AER (%)	ADDR (%)	AEC (J)	ET (s)
1	5.0	95.0	1.07 × 10^−7^	0.35
2	3.6	96.4	1.07 × 10^−7^	0.10
3	2.4	97.6	1.07 × 10^−7^	0.10
4	1.3	98.7	1.08 × 10^−7^	0.12
5	4.3	95.7	1.07 × 10^−7^	0.13
6	2.6	97.4	1.07 × 10^−7^	0.12
7	8.0	92.0	1.07 × 10^−7^	0.10
8	8.3	91.7	1.07 × 10^−7^	0.10
9	6.5	93.5	1.07 × 10^−7^	0.10
10	4.7	95.3	1.07 × 10^−7^	0.11
11	7.4	92.6	1.07 × 10^−7^	0.09
12	10.0	90.0	1.08 × 10^−7^	0.11
13	5.7	94.3	1.07 × 10^−7^	0.11
14	10.6	89.4	1.07 × 10^−7^	0.10
15	0.0	100.0	1.07 × 10^−7^	0.11
16	4.4	95.6	1.06 × 10^−7^	0.10
17	3.7	96.3	1.06 × 10^−7^	0.11
18	8.1	91.9	1.07 × 10^−7^	0.11
19	4.8	95.2	1.07 × 10^−7^	0.10
20	7.2	92.8	1.07 × 10^−7^	0.11
. . .	. . .	. . .	. . .	. . .
50	3.9	96.1	1.07 × 10^−7^	0.12
**Average**	**5.0**	**95.0**	**1.0688 × 10^−7^**	**0.1**

**Table 3:** Performance of the K-BCO Compared with Other Similar State-of-the-Art.

Algorithms	AER (%)	ADDR (%)	AEC (J)	ET (S)
H-LEACH	24.14	75.86	0.0237	0.03
DBCP	0.0991	72.07	1.973833	0.011
ABC-ACO	9.923	90.08	0.0799	0.083
K-BCO	5.0	95.0	1.0688 × 10^−7^	0.1

## Data Availability

Data are contained within the article.
